# 2,3-Diamino­pyridinium 3-amino­benzoate

**DOI:** 10.1107/S1600536809024362

**Published:** 2009-07-01

**Authors:** Kasthuri Balasubramani, Hoong-Kun Fun

**Affiliations:** a X-ray Crystallography Unit, School of Physics, Universiti Sains Malaysia, 11800 USM, Penang, Malaysia

## Abstract

In the title salt, C_5_H_8_N_3_^+^·C_7_H_6_NO_2_^−^, the pyridine N atom of the 2,3-diamino­pyridine mol­ecule is protonated. The proton­ated N atom and one of the two N atoms of the 2-amino groups are hydrogen bonded to the 3-amino­benzoate anion through a pair of N—H⋯O hydrogen bonds, forming an *R*_2_^2^(8) ring motif. The carboxyl­ate mean plane of the 3-amino­benzoate anion is twisted by 8.81 (7)° from the attached ring. The crystal structure is further stabilized by π–π inter­actions [centroid–centroid distance 3.6827 (7) Å].

## Related literature

For substituted pyridines, see: Pozharski *et al.* (1997 ▶); Katritzky *et al.* (1996 ▶). For hydrogen bonding in pyridine and its substituted derivatives, see: Jeffrey & Saenger (1991 ▶); Jeffrey (1997 ▶); Scheiner (1997 ▶). For related structures, see: Fun & Balasubramani (2009 ▶); Balasubramani & Fun (2009*a* ▶,*b* ▶). For bond-length data, see: Allen *et al.* (1987 ▶). For hydrogen-bond motifs, see: Bernstein *et al.* (1995 ▶). For the stability of the temperature controller used in the data collection, see: Cosier & Glazer (1986 ▶).
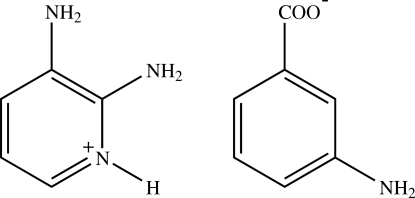


## Experimental

### 

#### Crystal data

C_5_H_8_N_3_^+^·C_7_H_6_NO_2_^−^
*M* *_r_* = 246.27
Monoclinic, 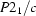

*a* = 9.9119 (2) Å
*b* = 10.1751 (2) Å
*c* = 12.4060 (2) Å
β = 106.811 (1)°
*V* = 1197.73 (4) Å^3^
*Z* = 4
Mo *K*α radiation
μ = 0.10 mm^−1^
*T* = 100 K
0.46 × 0.14 × 0.06 mm

#### Data collection

Bruker SMART APEXII CCD area-detector diffractometer
Absorption correction: multi-scan (*SADABS*; Bruker, 2005 ▶) *T*_min_ = 0.957, *T*_max_ = 0.994
19514 measured reflections
4434 independent reflections
2965 reflections with *I* > 2σ(*I*)
*R*_int_ = 0.046

#### Refinement

*R*[*F* ^2^ > 2σ(*F* ^2^)] = 0.050
*wR*(*F* ^2^) = 0.142
*S* = 1.06
4434 reflections
219 parameters
All H-atom parameters refined
Δρ_max_ = 0.33 e Å^−3^
Δρ_min_ = −0.23 e Å^−3^

### 

Data collection: *APEX2* (Bruker, 2005 ▶); cell refinement: *SAINT* (Bruker, 2005 ▶); data reduction: *SAINT*; program(s) used to solve structure: *SHELXTL* (Sheldrick, 2008 ▶); program(s) used to refine structure: *SHELXTL*; molecular graphics: *SHELXTL*; software used to prepare material for publication: *SHELXTL* and *PLATON* (Spek, 2009 ▶).

## Supplementary Material

Crystal structure: contains datablocks global, I. DOI: 10.1107/S1600536809024362/bt2977sup1.cif

Structure factors: contains datablocks I. DOI: 10.1107/S1600536809024362/bt2977Isup2.hkl

Additional supplementary materials:  crystallographic information; 3D view; checkCIF report

## Figures and Tables

**Table 1:** Hydrogen-bond geometry (Å, °)

*D*—H⋯*A*	*D*—H	H⋯*A*	*D*⋯*A*	*D*—H⋯*A*
N2—H2*N*2⋯O2^i^	0.91 (2)	2.02 (2)	2.9293 (14)	177.1 (17)
N3—H1*N*3⋯O2^i^	0.92 (2)	2.08 (2)	2.9854 (16)	167.3 (18)
N3—H2*N*3⋯O1^ii^	0.96 (2)	2.04 (2)	2.9544 (14)	158.1 (16)
N4—H1*N*4⋯O1^iii^	0.95 (2)	2.06 (2)	2.9794 (15)	162.3 (18)
N1—H1*N*1⋯O2	0.99 (2)	1.77 (2)	2.7510 (13)	167.1 (15)
N2—H1*N*2⋯O1	0.94 (2)	1.87 (2)	2.8086 (14)	175.7 (16)

Symmetry codes: (i) 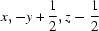; (ii) 
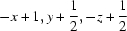
; (iii) 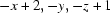.

**Fig. 1.**
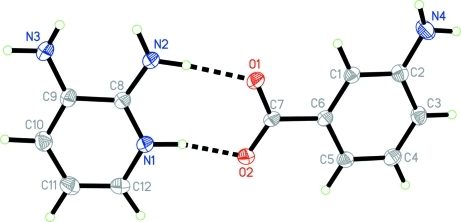
The molecular structure of (I), showing 50% probability displacement ellipsoids and the atom numbering scheme. Dashed lines indicate the hydrogen bonding.

**Fig. 2.**
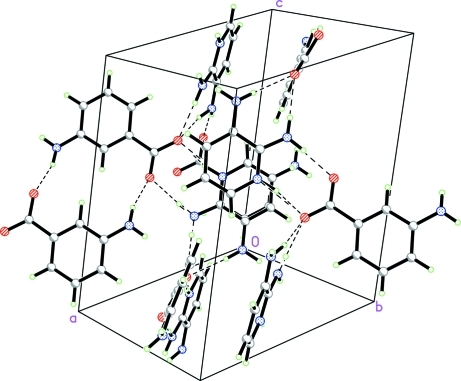
Part of the crystal packing of (I). Dashed lines indicate the hydrogen bonding.

## supplementary crystallographic information

### Comment

Pyridine and its derivatives play an important role in heterocyclic chemistry (Pozharski *et al.*, 1997; Katritzky *et al.*, 1996). Pyridine and its substituted derivatives are often involved in hydrogen-bond interactions (Jeffrey & Saenger, 1991; Jeffrey, 1997; Scheiner, 1997). The crystal structures of 2,3-diaminopyridinium 4-hydroxybenzoate (Fun & Balasubramani, 2009), 2,3-diaminopyridinium 4-nitrobenzoate (Balasubramani & Fun, 2009*a*) and 2,3-diaminopyridinium benzoate (Balasubramani & Fun, 2009*b*) have been reported by us recently. In the hope to study some interesting hydrogen-bonding interactions, the title compound (I) was synthesized. Its molecular and crystal structure is presented here.

The asymmetric unit of (I) (Fig 1), contains a protonated 2,3-diaminopyridinium cation and an 3-aminobenzoate anion. The bond lengths (Allen *et al.*, 1987) and angles are normal. In the 2,3-diaminopyridinium cation, the protonated N1 atom has lead to a slight increase in the C8—N1—C12 angle to 123.37 (11)°. The carboxylate group is twisted slightly from the ring with the dihedral angle between C1—C6 and O1/O2/C7/C6 planes being 8.81 (7)°. The 2,3-diaminopyridinium cation is planar, with a maximum deviation of 0.0126 (14) Å for atom C9.

In the crystal packing (Fig. 2), the protonated N1 atom and a nitrogen atom of the 2-amino group (N2) are hydrogen-bonded to the carboxylate oxygen atoms (O2 and O1) *via* a pair of N—H···O hydrogen bonds forming a ring motif *R*_2_^2^(8) (Bernstein *et al.*, 1995). The 2-amino groups (N2 and N3) are involved in N—H···O hydrogen bonding interactions to form a *R*^1^_2_(7) ring motif. The symmetry-related 3-aminobenzoate molecules are linked through N—H···O hydrogen-bonding to form a *R*^2^_2_(14) ring motif (Table 1 and Fig. 2). The cystal structure is further stabilized by a π-π stacking interaction between the aminopyridine rings (C8—C12/N1) with centroid-to-centroid distance of 3.6827 (7) Å, perpendicular interplanar distance of 3.3536 (5) Å.

### Experimental

Hot methanol solutions (20 ml) of 2,3-diaminopyridine (27 mg, Aldrich) and 3-aminobenzoic acid (35 mg, Merck) were mixed and warmed over a heating magnetic stirrer for 5 minutes. The resulting solution was allowed to cool slowly at room temperature. Crystals of (I) appeared from the mother liquor after a few days.

### Refinement

All the H atoms were located from the difference Fourier map and allowed to refine freely.

### Crystal data

**Table:**

C_5_H_8_N_3_^+^·C_7_H_6_NO_2_^−^	*F*(000) = 520
*M**_r_* = 246.27	*D*_x_ = 1.366 Mg m^−^^3^
Monoclinic, *P*2_1_/*c*	Mo *K*α radiation, λ = 0.71073 Å
Hall symbol: -P 2ybc	Cell parameters from 3436 reflections
*a* = 9.9119 (2) Å	θ = 2.9–32.6°
*b* = 10.1751 (2) Å	µ = 0.10 mm^−^^1^
*c* = 12.4060 (2) Å	*T* = 100 K
β = 106.811 (1)°	Plate, brown
*V* = 1197.73 (4) Å^3^	0.46 × 0.14 × 0.06 mm
*Z* = 4	

### Data collection

**Table:**

Bruker SMART APEXII CCD area-detector diffractometer	4434 independent reflections
Radiation source: fine-focus sealed tube	2965 reflections with *I* > 2σ(*I*)
graphite	*R*_int_ = 0.046
φ and ω scans	θ_max_ = 32.8°, θ_min_ = 2.2°
Absorption correction: multi-scan (*SADABS*; Bruker, 2005)	*h* = −14→15
*T*_min_ = 0.957, *T*_max_ = 0.994	*k* = −12→15
19514 measured reflections	*l* = −18→18

### Refinement

**Table:**

Refinement on *F*^2^	Primary atom site location: structure-invariant direct methods
Least-squares matrix: full	Secondary atom site location: difference Fourier map
*R*[*F*^2^ > 2σ(*F*^2^)] = 0.050	Hydrogen site location: inferred from neighbouring sites
*wR*(*F*^2^) = 0.142	All H-atom parameters refined
*S* = 1.06	*w* = 1/[σ^2^(*F*_o_^2^) + (0.0681*P*)^2^ + 0.0752*P*] where *P* = (*F*_o_^2^ + 2*F*_c_^2^)/3
4434 reflections	(Δ/σ)_max_ < 0.001
219 parameters	Δρ_max_ = 0.33 e Å^−^^3^
0 restraints	Δρ_min_ = −0.23 e Å^−^^3^

### Special details

**Table:**

Experimental. The crystal was placed in the cold stream of an Oxford Cryosystems Cobra open-flow nitrogen cryostat (Cosier & Glazer, 1986) operating at 100.0 (1) K.
Geometry. All e.s.d.'s (except the e.s.d. in the dihedral angle between two l.s. planes) are estimated using the full covariance matrix. The cell e.s.d.'s are taken into account individually in the estimation of e.s.d.'s in distances, angles and torsion angles; correlations between e.s.d.'s in cell parameters are only used when they are defined by crystal symmetry. An approximate (isotropic) treatment of cell e.s.d.'s is used for estimating e.s.d.'s involving l.s. planes.
Refinement. Refinement of F^2^ against ALL reflections. The weighted R-factor wR and goodness of fit S are based on F^2^, conventional R-factors R are based on F, with F set to zero for negative F^2^. The threshold expression of F^2^ > σ(F^2^) is used only for calculating R-factors(gt) etc. and is not relevant to the choice of reflections for refinement. R-factors based on F^2^ are statistically about twice as large as those based on F, and R- factors based on ALL data will be even larger.

### Fractional atomic coordinates and isotropic or equivalent isotropic displacement parameters (Å^2^)

**Table:**

	*x*	*y*	*z*	*U*_iso_*/*U*_eq_	
N1	0.45804 (11)	0.32315 (11)	0.46427 (8)	0.0219 (2)	
N2	0.56864 (12)	0.28026 (12)	0.32806 (9)	0.0252 (2)	
N3	0.36894 (13)	0.43692 (12)	0.17546 (9)	0.0275 (2)	
C8	0.46690 (12)	0.34222 (12)	0.35929 (10)	0.0199 (2)	
C9	0.36669 (13)	0.42721 (12)	0.28567 (10)	0.0223 (2)	
C10	0.26897 (14)	0.48893 (14)	0.32782 (11)	0.0276 (3)	
C11	0.26420 (14)	0.46612 (14)	0.43889 (12)	0.0286 (3)	
C12	0.35938 (13)	0.38245 (13)	0.50509 (11)	0.0252 (3)	
H10	0.1974 (16)	0.5508 (16)	0.2749 (13)	0.032 (4)*	
H11	0.1881 (16)	0.5151 (16)	0.4685 (13)	0.033 (4)*	
H12	0.3671 (16)	0.3657 (15)	0.5851 (13)	0.028 (4)*	
H1N1	0.5279 (18)	0.2611 (17)	0.5122 (14)	0.040 (5)*	
H1N2	0.6338 (18)	0.2303 (17)	0.3822 (15)	0.040 (5)*	
H2N2	0.5864 (19)	0.2985 (18)	0.2620 (16)	0.045 (5)*	
H1N3	0.453 (2)	0.4228 (19)	0.1593 (16)	0.056 (6)*	
H2N3	0.311 (2)	0.504 (2)	0.1305 (16)	0.053 (5)*	
C4	0.96875 (12)	−0.19646 (13)	0.66376 (10)	0.0226 (2)	
C5	0.89408 (12)	−0.08847 (12)	0.60585 (10)	0.0206 (2)	
H1	0.7131 (18)	−0.0025 (17)	0.7829 (14)	0.042 (5)*	
H2	0.8458 (18)	−0.1824 (17)	0.8828 (15)	0.039 (4)*	
H3	1.0060 (16)	−0.3052 (16)	0.8108 (13)	0.032 (4)*	
H5	0.9082 (15)	−0.0651 (14)	0.5321 (12)	0.023 (4)*	
C6	0.80059 (12)	−0.01887 (12)	0.64902 (9)	0.0191 (2)	
C7	0.72259 (12)	0.09741 (12)	0.58352 (9)	0.0198 (2)	
O1	0.75796 (9)	0.13487 (9)	0.49856 (7)	0.0232 (2)	
O2	0.62586 (10)	0.15115 (9)	0.61617 (7)	0.0259 (2)	
N4	1.05563 (14)	−0.26916 (14)	0.61717 (11)	0.0347 (3)	
C1	0.78080 (13)	−0.05510 (13)	0.75193 (10)	0.0231 (2)	
C2	0.85824 (14)	−0.16013 (13)	0.81132 (11)	0.0261 (3)	
C3	0.94998 (13)	−0.23049 (13)	0.76778 (11)	0.0249 (3)	
H1N4	1.097 (2)	−0.224 (2)	0.5680 (17)	0.057 (6)*	
H2N4	1.109 (2)	−0.329 (2)	0.6604 (17)	0.053 (5)*	

### Atomic displacement parameters (Å^2^)

**Table:**

	*U*^11^	*U*^22^	*U*^33^	*U*^12^	*U*^13^	*U*^23^
N1	0.0232 (5)	0.0234 (5)	0.0198 (5)	0.0005 (4)	0.0075 (4)	0.0013 (4)
N2	0.0283 (5)	0.0289 (6)	0.0206 (5)	0.0065 (4)	0.0105 (4)	0.0062 (4)
N3	0.0298 (6)	0.0314 (6)	0.0196 (5)	0.0039 (5)	0.0045 (4)	0.0048 (4)
C8	0.0208 (5)	0.0193 (5)	0.0196 (5)	−0.0018 (4)	0.0059 (4)	0.0005 (4)
C9	0.0225 (5)	0.0220 (6)	0.0204 (5)	−0.0007 (5)	0.0031 (4)	0.0010 (4)
C10	0.0238 (6)	0.0285 (7)	0.0283 (6)	0.0038 (5)	0.0041 (5)	0.0003 (5)
C11	0.0252 (6)	0.0307 (7)	0.0310 (7)	0.0016 (5)	0.0097 (5)	−0.0041 (5)
C12	0.0262 (6)	0.0280 (7)	0.0234 (6)	−0.0009 (5)	0.0104 (5)	−0.0029 (5)
C4	0.0201 (5)	0.0230 (6)	0.0257 (6)	−0.0015 (5)	0.0083 (4)	0.0014 (5)
C5	0.0209 (5)	0.0223 (6)	0.0187 (5)	−0.0014 (4)	0.0057 (4)	0.0012 (4)
C6	0.0200 (5)	0.0196 (5)	0.0171 (5)	−0.0019 (4)	0.0043 (4)	−0.0002 (4)
C7	0.0231 (5)	0.0207 (6)	0.0151 (5)	−0.0020 (4)	0.0046 (4)	−0.0015 (4)
O1	0.0251 (4)	0.0266 (5)	0.0183 (4)	0.0010 (4)	0.0072 (3)	0.0034 (3)
O2	0.0318 (5)	0.0272 (5)	0.0213 (4)	0.0078 (4)	0.0116 (4)	0.0033 (3)
N4	0.0351 (6)	0.0352 (7)	0.0405 (7)	0.0139 (5)	0.0216 (5)	0.0126 (6)
C1	0.0257 (6)	0.0234 (6)	0.0217 (6)	0.0010 (5)	0.0096 (5)	0.0016 (5)
C2	0.0302 (6)	0.0279 (7)	0.0223 (6)	0.0012 (5)	0.0107 (5)	0.0061 (5)
C3	0.0235 (6)	0.0243 (6)	0.0273 (6)	0.0016 (5)	0.0078 (5)	0.0067 (5)

### Geometric parameters (Å, °)

**Table:**

N1—C8	1.3445 (15)	C4—N4	1.3822 (17)
N1—C12	1.3650 (16)	C4—C3	1.3995 (17)
N1—H1N1	0.997 (18)	C4—C5	1.4013 (17)
N2—C8	1.3382 (16)	C5—C6	1.3907 (16)
N2—H1N2	0.936 (18)	C5—H5	0.994 (14)
N2—H2N2	0.906 (19)	C6—C1	1.3961 (16)
N3—C9	1.3775 (16)	C6—C7	1.5147 (17)
N3—H1N3	0.92 (2)	C7—O1	1.2621 (14)
N3—H2N3	0.96 (2)	C7—O2	1.2677 (14)
C8—C9	1.4296 (16)	N4—H1N4	0.95 (2)
C9—C10	1.3781 (18)	N4—H2N4	0.88 (2)
C10—C11	1.4115 (19)	C1—C2	1.3954 (18)
C10—H10	1.030 (16)	C1—H1	1.017 (17)
C11—C12	1.3569 (19)	C2—C3	1.3839 (18)
C11—H11	1.054 (16)	C2—H2	0.957 (17)
C12—H12	0.988 (15)	C3—H3	1.000 (16)
			
C8—N1—C12	123.37 (11)	N4—C4—C3	121.21 (12)
C8—N1—H1N1	116.1 (10)	N4—C4—C5	120.39 (11)
C12—N1—H1N1	120.5 (10)	C3—C4—C5	118.38 (11)
C8—N2—H1N2	118.0 (10)	C6—C5—C4	120.92 (11)
C8—N2—H2N2	121.7 (12)	C6—C5—H5	121.3 (8)
H1N2—N2—H2N2	119.1 (15)	C4—C5—H5	117.7 (8)
C9—N3—H1N3	118.6 (12)	C5—C6—C1	120.21 (11)
C9—N3—H2N3	116.7 (11)	C5—C6—C7	118.99 (10)
H1N3—N3—H2N3	114.2 (16)	C1—C6—C7	120.79 (11)
N2—C8—N1	118.57 (11)	O1—C7—O2	123.71 (11)
N2—C8—C9	122.86 (11)	O1—C7—C6	117.51 (10)
N1—C8—C9	118.57 (11)	O2—C7—C6	118.77 (10)
N3—C9—C10	123.98 (12)	C4—N4—H1N4	116.5 (12)
N3—C9—C8	118.02 (11)	C4—N4—H2N4	117.0 (12)
C10—C9—C8	117.90 (11)	H1N4—N4—H2N4	115.6 (17)
C9—C10—C11	121.41 (12)	C2—C1—C6	118.93 (12)
C9—C10—H10	117.8 (8)	C2—C1—H1	121.7 (10)
C11—C10—H10	120.8 (8)	C6—C1—H1	119.3 (10)
C12—C11—C10	118.71 (12)	C3—C2—C1	120.88 (12)
C12—C11—H11	121.8 (9)	C3—C2—H2	121.0 (10)
C10—C11—H11	119.4 (9)	C1—C2—H2	118.1 (10)
C11—C12—N1	120.00 (12)	C2—C3—C4	120.64 (12)
C11—C12—H12	123.7 (9)	C2—C3—H3	120.7 (9)
N1—C12—H12	116.1 (9)	C4—C3—H3	118.6 (9)
			
C12—N1—C8—N2	179.31 (11)	C4—C5—C6—C1	0.65 (18)
C12—N1—C8—C9	−1.04 (17)	C4—C5—C6—C7	179.83 (11)
N2—C8—C9—N3	5.26 (18)	C5—C6—C7—O1	−7.75 (16)
N1—C8—C9—N3	−174.37 (11)	C1—C6—C7—O1	171.43 (11)
N2—C8—C9—C10	−178.13 (12)	C5—C6—C7—O2	171.97 (11)
N1—C8—C9—C10	2.23 (17)	C1—C6—C7—O2	−8.85 (17)
N3—C9—C10—C11	174.31 (12)	C5—C6—C1—C2	1.28 (18)
C8—C9—C10—C11	−2.08 (19)	C7—C6—C1—C2	−177.89 (11)
C9—C10—C11—C12	0.7 (2)	C6—C1—C2—C3	−2.1 (2)
C10—C11—C12—N1	0.6 (2)	C1—C2—C3—C4	1.0 (2)
C8—N1—C12—C11	−0.41 (19)	N4—C4—C3—C2	−177.33 (13)
N4—C4—C5—C6	176.53 (12)	C5—C4—C3—C2	0.93 (19)
C3—C4—C5—C6	−1.75 (18)		

### Hydrogen-bond geometry (Å, °)

**Table:**

*D*—H···*A*	*D*—H	H···*A*	*D*···*A*	*D*—H···*A*
N2—H2N2···O2^i^	0.91 (2)	2.02 (2)	2.9293 (14)	177.1 (17)
N3—H1N3···O2^i^	0.92 (2)	2.08 (2)	2.9854 (16)	167.3 (18)
N3—H2N3···O1^ii^	0.96 (2)	2.04 (2)	2.9544 (14)	158.1 (16)
N4—H1N4···O1^iii^	0.95 (2)	2.06 (2)	2.9794 (15)	162.3 (18)
N1—H1N1···O2	0.99 (2)	1.77 (2)	2.7510 (13)	167.1 (15)
N2—H1N2···O1	0.94 (2)	1.87 (2)	2.8086 (14)	175.7 (16)

Symmetry codes: (i) *x*, −*y*+1/2, *z*−1/2; (ii) −*x*+1, *y*+1/2, −*z*+1/2; (iii) −*x*+2, −*y*, −*z*+1.
